# The Extravagance of Small Fever Hospitals

**Published:** 1898-03-12

**Authors:** 


### The Extravagance of Small Fever Hospitals.

One great reason why fever hospitals should not be unduly multiplied is the enormous increase of expense which is thereby entailed; another is that in proportion as such hospitals are numerous they are small, and small fever hospitals are not only wastefully extravagant both as concerns construction and up-keep, but are far less efficient as a means of isolating and treating the diseases in question than are hospitals of a fair size.

If the control of infectious disease in a district is to be undertaken in full seriousness, it is above all things necessary that the hospital should be always ready. We may question, and no doubt many of us do question very seriously, whether the isolation of infectious diseases which is provided by the fever hospitals in our large towns, has any very great influence in limiting their spread; but in the country it is different. There, by a fairly active enforcement of the Notification Act, it is possible to pick out first cases in a manner which is quite out of the question in more crowded centres of population, and thus to nip epidemics in the bud, if, that is, the hospital is kept in a state of constant readiness. The great vice, however, of small isolation hospitals is unpreparedness. The apparent wastefulness of keeping up a staff for an empty hospital, and the constant expense involved in maintaining all things in readiness for patients that never come, soon tell upon a small and maybe a poor sanitary authority, and "economies" are soon introduced, which quickly destroy the efficiency of the hospital as a means of dealing with sudden outbreaks and with first cases.

Again, it is absolutely essential that small-pox should have a hospital all to itself, while experience is tending more and more to show that diphtheria should not be mixed up in the same wards with scarlet fever. Every district then, before it can consider itself properly fitted up for the management of infectious diseases, must have two completely distinct hospitals, and in its fever hospital must have separate accommodation for at least two diseases.

Needless to say that this would be a tremendous strain upon districts whose population is small, and it is now very common for several districts to combine for hospital purposes, special provision being made with this object in the Isolation Hospitals Act. We think, however, that this system of combination should be carried out much more thoroughly and much more systematically than has hitherto been done, and more especially do we urge than that combinations should take place between urban and rural authorities. One of the first essentials for an isolation hospital in a rural district is that it shall be centrally placed near that point to which the main roads converge, and this is always at or near a town. On the other hand it is most desirable in many urban districts that the hospital should be placed outside the more crowded area, often actually in the contiguous rural district. Thus it happens that the natural place for isolation hospitals, both rural and urban, is in the neighbourhood of towns, and there seems no possible reason why the same hospitals should not be made to serve for both. Nevertheless, this obviously right course is too often not adopted. Take as an example what has lately happened at Croydon. The borough of Croydon some time ago erected a capital hospital at Waddon on an open, airy site at the very edge of, if not actually outside, the borough. And now the Croydon District Council is erecting another hospital just over the way at Beddington to serve for the nine parishes encircling the borough, which is their natural centre. Geographical and other considerations point to practically the same part as the most appropriate for hospital purposes, whether for the rural or the urban population; but instead of co-operating, the town sets up one hospital and the country another, each of which has to have its doctor, matron, porter, cook, its complete staff of day and night nurses for each class of disease, its diphtheria, scarlet fever, and observation wards, its dispensary, its laundry, its mortuary, its disinfecting apparatus, and all those other expensive appliances which go under the head of administration; and all for about thirty beds!

It is a terrible waste, which surely might have been prevented by a little foresight and a little exercise of the spirit of compromise. The same difficulty recently cropped up at Bedford. There the rural authorities were about to build an isolation hospital, and it seemed only reasonable that the town of Bedford, which badly wants some hospital accommodation for infectious diseases, should co-operate; but it refused. But many other places could be mentioned where, for lack of co-operation and of some foresight in plotting out the country into hospital districts, wasteful expenditure is being incurred, and a constant risk of inefficiency is being run.

